# Validation and Utilization of a Clinical Next-Generation Sequencing Panel for Selected Cardiovascular Disorders

**DOI:** 10.3389/fcvm.2017.00011

**Published:** 2017-03-15

**Authors:** Patrícia B. S. Celestino-Soper, Hongyu Gao, Ty C. Lynnes, Hai Lin, Yunlong Liu, Katherine G. Spoonamore, Peng-Sheng Chen, Matteo Vatta

**Affiliations:** ^1^Department of Medical and Molecular Genetics, Indiana University School of Medicine, Indianapolis, IN, USA; ^2^Center for Computational Biology and Bioinformatics, Indiana University Purdue University Indianapolis, Indianapolis, IN, USA; ^3^Division of Cardiology, Department of Medicine, Krannert Institute of Cardiology, Indiana University School of Medicine, Indianapolis, IN, USA

**Keywords:** next-generation sequencing, sequencing panels, cardiovascular, panel validation, clinical sequencing

## Abstract

The development of high-throughput technologies such as next-generation sequencing (NGS) has allowed for thousands of DNA loci to be interrogated simultaneously in a fast and economical method for the detection of clinically deleterious variants. Whenever a clinical diagnosis is known, a targeted NGS approach involving the use of disease-specific gene panels can be employed. This approach is often valuable as it allows for a more specific and clinically relevant interpretation of results. Here, we describe the customization, validation, and utilization of a commercially available targeted enrichment platform for the scalability of clinical diagnostic cardiovascular genetic tests, including the design of the gene panels, the technical parameters for the quality assurance and quality control, the customization of the bioinformatics pipeline, and the post-bioinformatics analysis procedures. Regions of poor base coverage were detected and targeted by Sanger sequencing as needed. All panels were successfully validated using genotype-known DNA samples either commercially available or from research subjects previously tested in outside clinical laboratories. In our experience, utilizing several of the sub-panels in a clinical setting with 33 real-life cardiovascular patients, we found that 20% of tests requested were reported to have at least one pathogenic or likely pathogenic variant that could explain the patient phenotype. For each of these patients, the positive results may aid the clinical team and the patients in best developing a disease management plan and in identifying relatives at risk.

## Introduction

In the clinical genetics setting, most deleterious DNA variants can be detected by DNA sequencing. The development of high-throughput technologies such as next-generation sequencing (NGS) has allowed for thousands of target regions to be interrogated in a fast and economical approach, when compared to the more traditional technique of Sanger sequencing. Different NGS approaches such as whole-genome sequencing (WGS) and whole-exome sequencing (WES) have been employed especially for gene discovery. In particular, WGS and WES can be used as clinical testing modalities when a clinical diagnosis cannot be unequivocally established or for genetic disorders for which no other established clinical testing is available. However, when a clinical diagnosis has been reached, a more targeted NGS approach involving the use of comprehensive disease-specific gene panels can be employed. In the clinical setting for example, gene panels may be designed to target genes associated with a disease or a group of related diseases depending on the level of complexity of clinical and phenotypic overlap. This approach is often valuable as it allows for a more specific and clinically relevant interpretation of results with variants in genomic loci *a priori* selected for their disease association. Additionally, when compared to WGS and WES, gene panels have the practical benefit of having more robust sequence coverage of target loci, lower cost, and faster turnaround time (1, 2).

Here, we describe the customization, clinical validation, and utilization of a commercial NGS panel, the TruSight One (TSO) panel developed by Illumina, Inc., which targets the coding regions of 4,813 genes associated with human disease, enriching for over 62,000 exons and their splice sites. Although NGS is currently considered to be a well-established technique, the clinical validation of recently available commercial kits still remains a constant challenge and a necessary step to ensure the high quality of clinical practice. Here, we show that the method of choice was technically reliable for sequencing and base calling, and that the annotation and filtering methods selected from the literature successfully detected variants in the targeted regions. Target regions were enriched and captured using the Illumina Nextera TSO Enrichment Kit and sequenced using solid-state sequencing-by-synthesis technology employing the Illumina MiSeq desktop sequencer system. The sequencing data were processed using an in-house custom bioinformatics pipeline with variant calls generated using the Burrows–Wheeler Aligner (BWA) followed by GATK analysis, which generated a variant call format (.vcf) file to be used for final interpretation. We subdivided the TSO panel into six smaller panels for testing of the exonic and splicing regions of genes associated with cardiovascular diseases according to disease phenotype, including arrhythmogenic right ventricular cardiomyopathy (ARVC), dilated cardiomyopathy/left ventricular non-compaction (DCM/LVNC), hypertrophic cardiomyopathy (HCM), Marfan syndrome/Loeys–Dietz syndrome (MFS/LDS), thoracic aortic aneurysms and dissections (TAAD), and a comprehensive cardiomyopathy (CMP) panel. Splitting into sub-panels allows for the proper test requisition by the physician while minimizing the risk of incidental findings and the presence of confounding variants. Several sub-panels were designed to have overlapping genes. In addition, the large CMP panel allows physicians to request the sequencing of genes in a more a comprehensive approach, usually when the clinical presentation is not very predictive of a particular type of CMP. The performance for each sub-panel was established after bioinformatics analyses which detected regions of poor coverage. These regions were targeted by Sanger sequencing as needed. Overall, all panels were successfully validated using a series of available genotype-known samples. We also describe our experience utilizing several of the sub-panels in a clinical setting with a group of 33 real-life cardiovascular patients (35 NGS tests requested). In conclusion, the utilization of the validated TSO sub-panels has provided us with a method to efficiently and economically search for thousands of clinically significant variants in one single experiment. Given the success of this project, we aim to continue the validation of additional sub-panels for other human disorders.

## Materials and Methods

### NGS Cardiovascular Panels

The commercial TSO panel consisted of all 4,813 genes. We subdivided the gene content of the TSO panel into six clinical NGS panels which were further validated. The six clinical NGS panels made available for ordering of clinical testing each comprised of the sequencing of all coding regions and the immediate flanking regions of each exon of a specific group of genes. The CMP and the TAAD panels were also made available as reflex options (per physician request) when negative results were reported. Table 1 describes each validated panel and the genes they cover. Table S1 in Supplementary Material contains the gene symbol, gene name, genomic coordinates, and main gene transcript for each gene that was sequenced using one of the NGS panels.

**Table 1:** **Cardiovascular genetics next-generation sequencing (NGS) panel gene content**.

NGS sub-panel	Number of genes	Genes covered
Comprehensive cardiomyopathy (CMP) and reflex CMP	61	*ABCC9, ACTC1, ACTN2, ANKRD1, BAG3, CASQ2, CAV3, CRYAB, CSRP3, CTNNA3, DES, DMD, DSC2, DSG2, DSP, DTNA, EMD, EYA4, FHL1, FHL2, FKTN, GATAD1, GLA, JPH2, JUP, KLF10, LAMA4, LAMP2, LDB3, LMNA, MYBPC3, MYH6, MYH7, MYL2, MYL3, MYLK2, MYOZ2, NEBL, NEXN, PDLIM3, PKP2, PLN, PRKAG2, PSEN1, PSEN2, RAF1, RBM20, RYR2, SCN5A, SGCD, TAZ, TCAP, TMEM43, TMPO, TNNC1, TNNI3, TNNT2, TPM1, TTN, TTR, VCL*
Hypertrophic cardiomyopathy	18	*ACTC1, ACTN2, CSRP3, GLA, LAMP2, MYBPC3, MYH7, MYL2, MYL3, MYOZ2, NEXN, PLN, PRKAG2, TNNC1, TNNI3, TNNT2, TPM1, TTR*
Dilated cardiomyopathy	33	*ABCC9, ACTC1, ACTN2, ANKRD1, BAG3, CRYAB, CSRP3, DES, DMD, DTNA, EMD, EYA4, GATAD1, LAMP2, LDB3, LMNA, MYBPC3, MYH7, NEXN, PLN, RAF1, RBM20, SCN5A, SGCD, TAZ, TCAP, TNNC1, TNNI3, TNNT2, TPM1, TTN, TTR, VCL*
Arrythmogenic right ventricular cardiomyopathy	8	*CTNNA3, DSC2, DSG2, DSP, JUP, LMNA, PKP2, TMEM43*
Marfan syndrome and Loeys–Dietz syndrome	3	*FBN1, TGFBR1, TGFBR2*
Thoracic aortic aneurysms and dissections (TAAD) and reflex TAAD	18	*ACTA2, CBS, COL3A1, COL5A1, COL5A2, ELN, FBN1, FBN2, MED12, MYH11, MYLK, PLOD1, SLC2A10, SMAD3, SMAD4, TGFB2, TGFBR1, TGFBR2*

### Validation Samples

The validation samples consisted of 23 genotype-known and 3 genotype-unknown samples (Table S2 in Supplementary Material). These samples were tested for their ability to result in successful libraries and sequence runs, as well as for evaluation and validation of the current NGS panel. DNA extraction methodology for each sample is listed in Table S2 in Supplementary Material. Validation samples were anonymous Coriell gDNA specimens or clinical samples that have been recruited through and Indiana University IRB-approved Genetic Registry Specimen Repository with minimal description in the report and no listed identifiable information.

### Patient Samples

Patient samples consisted of clinical cases sent to the Indiana University School of Medicine Molecular Genetics Diagnostic Laboratory of the Department of Medical and Molecular Genetics, Indiana University School of Medicine, in the period of January 2015 to December 2015, with requisition for testing with one the following cardiovascular NGS panels: ARVC, DCM/LVNC, HCM, MFS/LDS, TAAD, or CMP. Patient samples listed in this manuscript have been described without identifiable information. All patient DNAs used for testing were extracted from whole blood collected in purple-top tube using the Qiagen’s Gentra Puregene Blood Kit (Qiagen, Germantown, MD, USA) following the manufacturer’s instructions. Samples were de-identified and only information about the NGS test requested, and variants identified were retained for the purpose of analysis of results for this manuscript.

### Validation Runs

Table S3 in Supplementary Material summarizes the scheme used to prepare libraries using Illumina’s TSO panel as specified by the standard and operating procedure (SOP). Each kit had reagents available for three runs. A repeat of NA12878 within a run was used for intra-run variability studies. A repeat of NA12878 between runs was used for inter-run variability studies. Additionally, different operators performed the tests according to the established technical protocols and SOP guidelines to allow for the evaluation of runs from libraries prepared by different operators (A or B). Furthermore, inter-lot, inter-day, and inter-run variability were assessed from runs of NA11931 between TSO_009 and TSO_013, and runs of NA11829 between TSO_010 and TSO_014. Single individuals (1-plex) or pools of three individuals (3-plex) were sequenced per flow-cell.

### Library Preparation and Sequence Data Generation

The Illumina TSO NGS panel was developed by Illumina, Inc. (San Diego, CA, USA) and includes over 125,395 80-mer probes that were designed against the human NCBI GRCh37/hg19 reference genome assembly. Information about the expected performance, targeted regions, content design, and other information can be found in the TSO full gene list, TSO Data Sheet, and TSO Technical Note in the manufacturer’s website.

Optimization and validation experiments were set up manually following the manufacturer’s instructions. Experiments were performed loading either a single sample (1-plex) or three samples (3-plex) per MiSeq run per flow-cell. After quantitation using Qubit (Thermo Fisher Scientific, Waltham, MA, USA), genomic DNA underwent Nextera tagmentation, which converts input genomic DNA into adapter-tagged libraries. Next, libraries were denatured and biotin-labeled probes specific to the targeted region were used for hybridization. The pool was then enriched for the targeted regions by adding streptavidin beads that bind to the biotinylated probes. Biotinylated DNA fragments bound to the streptavidin-coated beads were magnetically pulled down from the solution. The enriched DNA fragments were then eluted from the beads and hybridized for a second capture. Library preparation underwent quality control (QC) using an Agilent TapeStation, which was employed before library preparation and Qubit quantitation after library preparation. These steps provided the necessary metrics to assess the efficiency of fragmentation within the desired size range and the successful adapters/barcoding addition to each sample’s DNA fragment. Prepared libraries were then loaded on to a flow-cell for sequencing with the Illumina MiSeq desktop sequencer system, which acquired sequencing data points and generated a bam and a fastq file for sequence reads. The resulting sequence data were submitted to analysis if the data passed the acceptance and rejection criteria for analytic runs according to manufacturer’s instruction.

### Bioinformatics Pipeline

To analyze and characterize data generated from targeted re-sequencing, the following softwares were implemented in our bioinformatics pipeline: Trim Galore (version 0.3.2) to remove adaptor sequences and low quality reads; BWA (version 0.7.5a) (3) to align reads to human reference genome UCSC GRCh37/hg19; GenomeAnalysisTK-2.8-1 (4, 5) for local realignment, base quality recalibration, and variants identification; SAMtools (version 0.1.19) (6) and picard-tools-1.105 (http://picard.sourceforge.net) to manipulate alignment files; VCFtools (version 0.1.10) (7) and BEDTools (8) (version 2.17.0) to further process resulted variant VCF files; ANNOVAR (revision 529) (9) for annotating variants; and Human Gene Mutation Database (HGMD) (10–12) professional used for further characterizing variants. R (13) and PERL were used for additional data analysis and characterization. All data processing steps were compiled into an all-steps-in-one bash script. Running scripts and parameters applied are available at http://compbio.iupui.edu/group/6/pages/clinicalsequencing. Additionally, our target file, the TruSight One Sequencing Panel Manifest downloaded from Illumina, can be found at: support.illumina.com/content/dam/illumina-support/documents/downloads/productfiles/trusight/trusight-one-manifest-may-2014.zip.

Following these procedures generated a final report of variants from targeted gene regions. The report consisted of variant mapping information, gene annotation, amino acid change annotation (synonymous or non-synonymous variants), variant functional annotation [SIFT, PolyPhen, LRTs, and MutationTaster (14–20)], variant evolutionary conservation annotation [PhyloP and GERP++ (21–23)], variant presence and allele frequencies in currently publicly sequenced populations (dbSNP identifiers, 1000 Genomes Project allele, NHLBI-ESP 6500 exome project), and known disease-related functional annotation from the HGMD database. Quality parameters such as variant quality, read depth, mapping quality, and fisher strand bias were included in the final variants report as well (see Table S4 in Supplementary Material). Although the TSO panel includes 4,813 genes, in the clinical setting, only the genes included in the panel requested by the referring physician, genetic counselor, or other appropriate health care provider were analyzed and only variants for the requested panel were available for post-bioinformatics analyses of variants. Further information regarding the bioinformatics pipeline, can be found in the Supplementary Material.

### Post-Bioinformatics Analyses

The TSO sequencing panel was first tested in 3-plex experiments (three individuals pooled per flow-cell run), as specified by the manufacturer. Validation of coverage and SNP performance was completed using 3-plex and 1-plex runs. Variants found in the validation samples were compared to secondary data as specified in Table S2 in Supplementary Material for concordance and evaluation of several metrics including false positive (FP) and false negative (FN) rates, analytic sensitivity, analytic specificity, overall genotype concordance (OGC), non-reference sensitivity (NRS), non-reference discrepancy (NRD), non-reference genotype concordance (NRGC), and precision.

Samples received for clinical testing were run as 1-plex or 3-plex NGS panel experiments (panel selection as requested for each patient). Sanger (BigDye) sequencing was used to provide data for bases with insufficient coverage in exonic and splicing (±2 nucleotides from the exon) regions of genes of interest in the NGS panel run (<15× or <10× sequence depth, as needed). Several regions were recurrently found to have lower than 15× sequence depth in 1-plex validation runs (Table S5 in Supplementary Material) and were included in the default Sanger sequencing for clinical testing for each selected panel. “Products were sequenced using an Applied Biosystems 3500 xl Genetic Analyzer in conjunction with the ABI BigDye^®^ Terminator v3.1 Cycle Sequencing kit chemistry and protocol (ABI, Foster City, CA). Sequences where aligned to each gene and analyzed using Mutation Surveyor software V4.0.7 (SoftGenetics, State College, PA).” The limitations of the Sanger sequencing method are that the presence of DNA structural rearrangements (such as the deletion of an exon or multiple exons) may not be detected by sequence analysis. Additional tests analyzing DNA structural rearrangements should be recommended to those patients who are negative for sequencing analysis. Additionally, variants that may be found within known segmental duplication (SegDup) regions listed in Table S6 in Supplementary Material cannot be amplified and sequenced unambiguously by PCR and BigDye sequence, and therefore cannot be reported. Variants found outside those loci listed in Table S6 in Supplementary Material were attempted to be confirmed unambiguously by PCR and BigDye sequencing if they were classified as pathogenic/likely pathogenic or variant of uncertain clinical significance (VUS).

Variants found in clinical test samples were evaluated for their clinical effect as being pathogenic, likely pathogenic, gene modifier, VUS, likely benign, or benign as explained in the Supplementary Material (special cases may differ from the classification procedures). The first step included separating the variants based on their presence or absence in the HGMD database, in order to facilitate the review process, since the HGMD database provides some curation for variants with known disease association. Variants deemed to be pathogenic/likely pathogenic or VUS were confirmed by Sanger sequencing as deemed necessary by the laboratory director on a case-by-case basis. In our post-bioinformatics analysis, we have mostly adhered to the current American College of Medical Genetics and Genomics (ACMG) guidelines for the standard interpretation of genetic variants (24). However, some parameters such as frequency have been adapted to reflect the current knowledge about the increased complex inheritance pattern in several cardiac syndromes, previously regarded as pure monogenic Mendelian diseases such as dilated cardiomyopathy (DCM), HCM, and ARVC, in which 5–10% of cases can present with two or more deleterious variants (25).

## Results

### Panel Validation

Six NGS panels comprising of a select group of genes from the Illumina, Inc. TSO panel was optimized and validated using our in-house bioinformatics approach as described in Section “Materials and Methods.” Table 2 summarizes the metrics of the validation studies. Overall, the validation data for the NGS TSO and the cardiovascular sub-panels gave consistent and accurate genotype calls. A more detailed explanation of the validation results found in Table 2 is presented below.

**Table 2:** **Summary of validation for next-generation sequencing TruSight One (TSO) and cardiovascular sub-panels**.

1-Plex experiment (average of experiments ± SD)							
Metrics^b^	TSO panel (full)	Comprehensive cardiomyopathy (CMP) and reflex	Hypertrophic cardiomyopathy (HCM)	Dilated cardiomyopathy/left ventricular non-compaction (DCM/LVNC)	Arrhythmogenic right ventricular cardiomyopathy (ARVC)	Marfan syndrome/Loeys–Dietz syndrome (MFS/LDS)	Thoracic aortic aneurysms and dissections (TAAD) and reflex
Target region mean depth (*x*)	301.25 ± 35.56	309 ± 37.7	277 ± 30.38	322 ± 39.71	319.75 ± 43.22	240.25 ± 28.55	292.75 ± 32.84
Fraction of regions target depth ≥15×	0.97 ± 0.00	0.99 ± 0.00	1.00 ± 0.00	0.99 ± 0.00	0.99 ± 0.00	0.99 ± 0.00	0.99 ± 0.00
Accuracy	100%	100%	100%	100%	100%	100%	100%
Analytical sensitivity	0.96 ± 0.01	0.96 ± 0.02	1.00 ± 0.00	0.95 ± 0.03	0.98 ± 0.04	1.00 ± 0.00	0.96 ± 0.03
Analytical specificity	0.89 ± 0.03	0.93 ± 0.03	0.92 ± 0.04	0.93 ± 0.03	0.91 ± 0.05	1.00 ± 0.00	0.99 ± 0.02
False negative (FN) SNP rate	0.04 ± 0.01	0.04 ± 0.02	0.00 ± 0.00	0.05 ± 0.03	0.02 ± 0.04	0.00 ± 0.00	0.04 ± 0.03
False positive (FP) SNP rate	0.11 ± 0.03	0.07 ± 0.03	0.08 ± 0.04	0.07 ± 0.03	0.09 ± 0.05	0.00 ± 0.00	0.01 ± 0.02
Overall genotype concordance (OGC) range	0.81–0.94	0.82–0.94	0.83–1.00	0.81–0.97	0.81–1.00	N/A^a^	0.87–1.00
Non-reference sensitivity (NRS) range	0.91–0.98	0.87–1.00	0.94–1.00	0.86–1.00	0.81–1.00	N/A^a^	0.89–1.00
Non-reference discrepancy (NRD) range	0.07–0.20	0.06–0.19	0.00–0.17	0.04–0.20	0.00–0.19	N/A^a^	0.00–0.13
Non-reference genotype concordance (NRGC) range	0.90–0.97	0.87–1.00	0.94–1.00	0.86–1.00	0.81–1.00	N/A^a^	0.89–1.00
Precision range	0.85–0.97	0.86–0.98	0.85–1.00	0.87–1.00	0.81–1.00	N/A^a^	0.95–1.00
Number of loci with >15× coverage (length in basepairs)^c^	Not assessed	34 (3,329 bp)	3 (221 bp)	21 (1,561 bp)	6 (423 bp)	1 (100 bp)	6 (787 bp)
Number of SegDups loci (length in basepairs)^d^	Not assessed	5 (1,914 bp) 2^e^ (197 bp)	0 (0 bp)	2 (208 bp) 0^e^ (0 bp)	2 (197 bp)	0 (0 bp)	6 (1,917 bp) 1^e^ (173 bp)

**3-Plex experiment (average of experiments ± SD)**							
**Metrics^b^**	**TSO panel**	**CMP and CMP reflex**	**HCM**	**DCM/LVNC**	**ARVC**	**MFS/LDS**	**TAAD and TAAD reflex**

Target region mean depth (*x*)	117.48 ± 11.92	118.03 ± 10.42	108.41 ± 12.01	122.66 ± 10.57	124.07 ± 11.58	94.48 ± 9.75	114.76 ± 11.74
Fraction of regions target depth ≥15×	0.95 ± 0.01	0.98 ± 0.00	0.98 ± 0.01	0.98 ± 0.00	0.97 ± 0.01	0.98 ± 0.01	0.98 ± 0.00
Accuracy (%)	100	100	100	100	100	100	100
Analytical sensitivity	0.96 ± 0.01	0.96 ± 0.03	0.99 ± 0.02	0.94 ± 0.03	0.98 ± 0.04	0.97 ± 0.09	0.97 ± 0.04
Analytical specificity	0.89 ± 0.03	0.93 ± 0.03	0.88 ± 0.03	0.94 ± 0.03	0.91 ± 0.06	0.98 ± 0.06	0.96 ± 0.04
FN SNP rate	0.04 ± 0.01	0.04 ± 0.03	0.01 ± 0.02	0.06 ± 0.03	0.02 ± 0.04	0.03 ± 0.09	0.03 ± 0.04
FP SNP rate	0.11 ± 0.03	0.07 ± 0.03	0.12 ± 0.08	0.06 ± 0.03	0.09 ± 0.06	0.02 ± 0.06	0.04 ± 0.04
OGC range	0.81–0.94	0.82–0.94	0.83–1.00	0.81–0.97	0.81–1.00	N/A^a^	0.87–1.00
NRS range	0.91–0.98	0.87–1.00	0.94–1.00	0.86–1.00	0.81–1.00	N/A^a^	0.89–1.00
NRD range	0.07–0.20	0.06–0.19	0.00–0.17	0.04–0.20	0.00–0.19	N/A^a^	0.00–0.13
NRGC range	0.90–0.97	0.87–1.00	0.94–1.00	0.86–1.00	0.81–1.00	N/A^a^	0.89–1.00
Precision range	0.85–0.97	0.86–0.98	0.85–1.00	0.87–1.00	0.81–1.00	N/A^a^	0.95–1.00

*^a^Quantitative measurements OGC, NRS, NRD, NRGC, and precision were not computed for the MFS/LDS panel since only three variants were available for these analyses, and there was not enough power for the performance of the quantitative measurements above. Qualitative measurements of quality control for library preparation, sequencing performance, coverage, and SNP performance indicated that the variability among the runs listed above were within acceptable range*.

*^b^Samples MotherLP and ProbandJP were not used in the calculations of FN SNP rate, FP SNP rate, analytic sensitivity, analytic specificity, OGC, NRS, NRD, NRGC, and precision range. Only GenReg samples were used in accuracy calculations*.

*^c^Number of loci (with corresponding length in basepairs in parenthesis) that presented with lower than 15× coverage after 1-plex runs*.

*^d^Number of loci (with length in basepairs in parenthesis) that mapped to a region of known segmental duplications (SegDups)*.

*^e^Number of loci (with corresponding number of basepairs in parenthesis) with known SegDup that were successfully validated to be unambiguously amplified and sequenced out of the total regions and corresponding basepairs listed*.

#### Coverage

**Figure 1:**
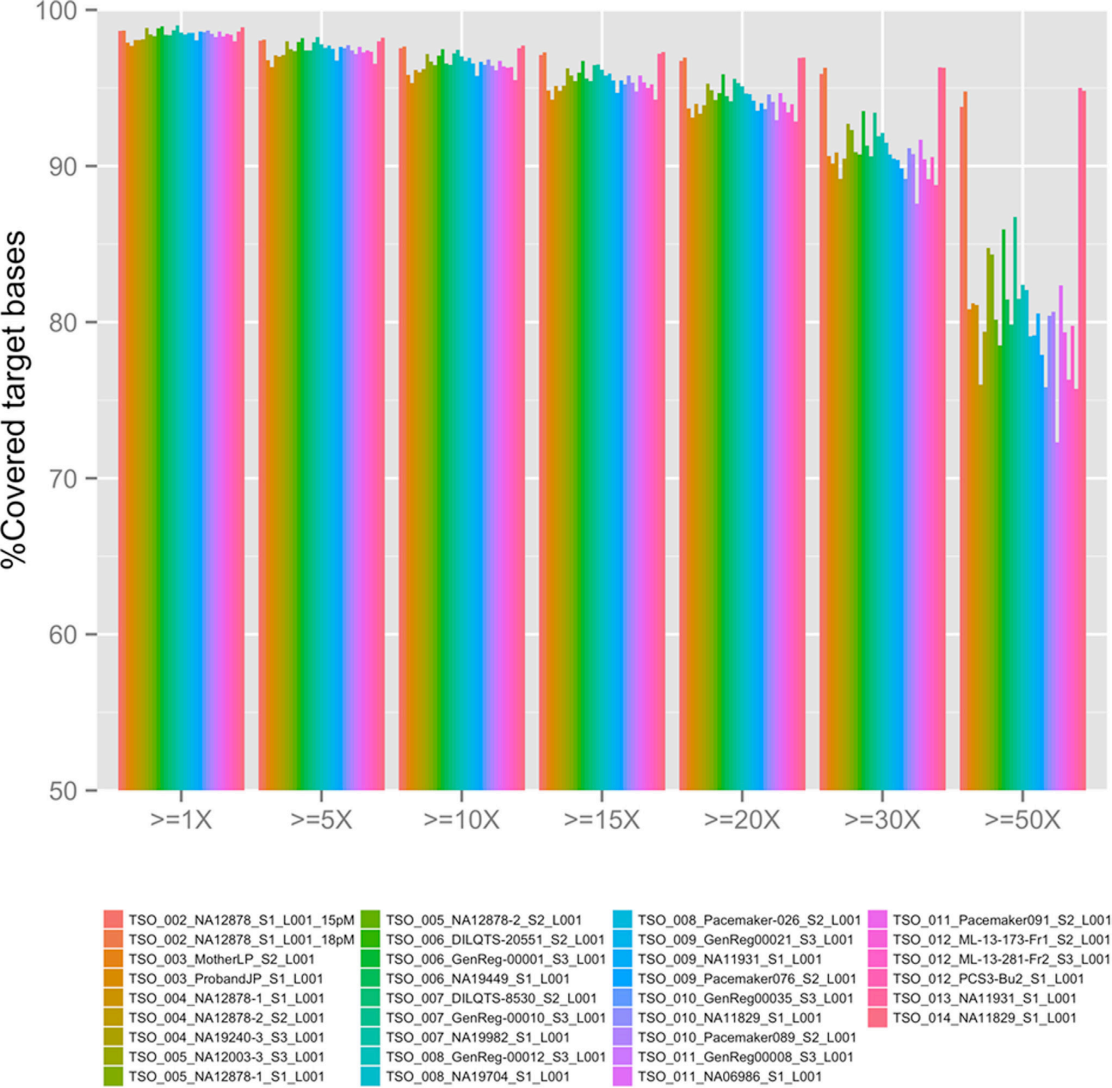
**TruSight One (TSO) next-generation sequencing depth in all validation runs**. Sequencing depth, *x*.

The overall coverage (sequence depth) of target bases for the TSO panel (see Figure 1) was dependent on the concentration of final library used in the sequencing run (compare TSO_002_NA12878 15 versus 18pM runs) as well as on the final number of samples pooled per sequencing run (compare for example, NA11829 in 3-plex run TSO_010 and in 1-plex run TSO_014). The same was true for all cardiovascular sub-panels as summarized on Table 2. Overall, better coverage was obtained from higher concentration of library used in the run and in 1-plex experiments (as was found later for patient runs). Regions of systematic low coverage were often found to fall within the first exon of the targeted genes, likely due to high GC content in these regions, which may affect probe binding. Other factors, such as an inherent suboptimal performance of certain capture probes, may also play a role in the decreased coverage of some regions; however, since the TSO panel is a commercial off-the-shelf product, we were not given the choice to add custom optimized probes to mitigate this problem. Regions of less than 15× depth of coverage from 1-plex experiments were selected for BigDye (Sanger) sequencing validation for each panel (number and length of loci are listed in Table 2). Loci pertaining to regions of known SegDups for each panel were also attempted to be validated. It is possible that additional loci may need to be Sanger sequenced after NGS testing of a given patient (for example, for confirmation or for testing of additional regions with low coverage or within SegDups).

#### Accuracy

De-identified DNA samples with various genotypes previously tested at an independent clinical laboratory were assayed. The assay showed complete concordance with expected results for all panels, following a blinded analysis. These results show validation of the TSO panel (and its sub-panels), of the bioinformatics pipeline, and of the post-bioinformatics filtering of variants. In addition, using a 1-plex run with NA12878 (TSO_002_18pM run), we determined the maximum length of indels properly detected by the TSO panel to be of 22 nucleotides in a homozygous state, and 30 nucleotides in a heterozygous state (both cases with satisfactory quality and sequence depth; see indel information in Materials and Methods in Supplementary Material).

#### Analytical Sensitivity, Analytical Specificity, FN Rates, and FP Rates

Table 2 lists the analytical sensitivity, analytical specificity, FN rates, and FP rates obtained in 1-plex and 3-plex NGS validation experiments when the data obtained (from a run, prior to BigDye confirmation) were compared to data from an outside source. Samples MotherLP and ProbandJP were not used in these calculations. Overall, very similar analytical sensitivity, analytical specificity, FN rates, and FP rates were obtained between 1-plex and 3-plex experiments.

BigDye confirmation was performed to test the FP and FN variants found in 1-plex experiments. Following BigDye confirmation, the results for 1-plex experiments shown in Table 2 were corrected to reflect the final analytical sensitivity, analytical specificity, and FN and FP rates for the exonic and splicing targeted regions that obtained sequence depth of ≥15× of genes in the six NGS panels (Table 3). The values in Table 3 represent the true expected reportable performance of the six NGS panels for 1-plex runs (since exonic and splicing regions of genes with <15× or <10× sequence depth were covered by BigDye sequencing, as deemed necessary). With an average of 1, 0.996, 0, and 0.004 for the sensitivity, specificity, FN rate, and FP rate, respectively, our panels demonstrated an excellent performance for the clinical application.

**Table 3:** **Corrected SNP performance validation in 1-plex comprehensive comprehensive cardiomyopathy (CMP) panel validation after BigDye sequencing**.

Metrics	TruSight One panel	CMP and reflex	Hypertrophic cardiomyopathy	Dilated cardiomyopathy/left ventricular non-compaction	Arrhythmogenic right ventricular cardiomyopathy	Marfan syndrome/Loeys–Dietz syndrome	Thoracic aortic aneurysms and dissections and reflex
Analytical sensitivity	N/A^a^	1.00 ± 0.00	1.00 ± 0.00	1.00 ± 0.00	1.00 ± 0.00	N/A^a^	1.00 ± 0.00
Analytical specificity	N/A^a^	0.99 ± 0.00	1.00 ± 0.00	0.99 ± 0.00	1.00 ± 0.00	N/A^a^	1.00 ± 0.00
False negative (FN) SNP rate	N/A^a^	0.00 ± 0.00	0.00 ± 0.00	0.00 ± 0.00	0.00 ± 0.00	N/A^a^	0.00 ± 0.00
False positive (FP) SNP rate	N/A^a^	0.01 ± 0.00	0.00 ± 0.00	0.01 ± 0.00	0.00 ± 0.00	N/A^a^	0.00 ± 0.00

*^a^Measurements not calculated for the designated panels (no BigDye sequencing performed)*.

#### Assay Precision

The overall precision of each panel was calculated by running three different samples various times. Runs were compared to secondary data available (Illumina Platinum Genomes and 1000 G project) and also to a series of repeated runs in our laboratory. The repeatability was tested by the intra-run variability (two libraries of the same starting genomic DNA sample run twice in the same sequencing experiment), while the reproducibility was tested by the inter-run variability (two libraries of the same starting genomic DNA sample run twice in the separate sequencing experiments). Additionally, inter-operator variability was tested by allowing operator A to perform experiment TSO_004 while operator B performed experiment TSO_005. Furthermore, inter-lot, inter-day, and inter-run variability were assessed from runs of NA11931 between TSO_009 and TSO_013, and runs of NA11829 between TSO_010 and TSO_014. Several measurements were used to assess variability. The OGC, NRS, NRD, and NRGC were computed as previously published (26, 27). OGC, NRS, NRD, and NRGC were calculated treating each replicate alternatively as comparison set and evaluation set. Precision was calculated as True Positive SNPs divided by SNPs obtained from the Miseq run. Table 2 describes the range of each measurement for both MiSeq runs compared to secondary data and to MiSeq repeated runs. Overall, values obtained from comparing our TSO NGS experiments to other methods used by secondary testing sites showed more variability than when comparing to our repeated runs (Table S7 in Supplementary Material).

#### Assay Robustness

DNAs obtained from different sources (whole blood, cell lines, buccal swabs, and frozen post-mortem blood) passed all QC steps from DNA extraction to library preparation, to required MiSeq sequencing metrics. Only one MiSeq instrument is available in our molecular genetics laboratory. Table S2 in Supplementary Material summarizes the various runs performed and Table 2 summarizes the precision obtained under various conditions. It is evident that this assay is sufficiently robust to accommodate variations among consumables, technologists, and origin of DNA. However, assessment of the impact of contaminants on the performance of the test has not been systematically performed.

### Patient Testing

**Figure 2:**
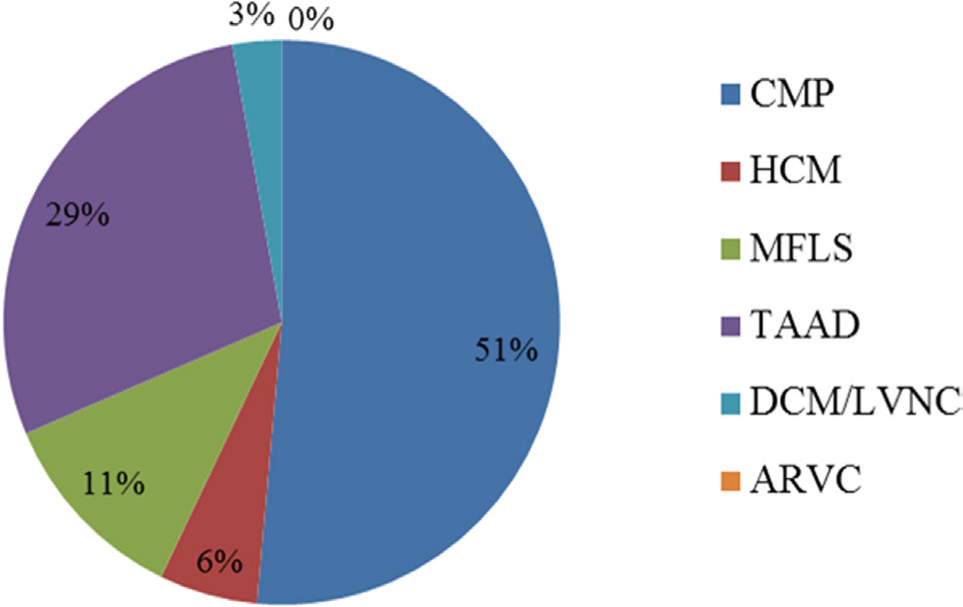
**Distribution of panels ordered for patient testing (%)**. The ARVC panel was not ordered for patient testing during the time-frame selected. Two patients (IDs 24 and 32) had sequencing reported for two panels (CMP as a reflex). ARVC, arrhythmogenic right ventricular cardiomyopathy panel; CMP, comprehensive cardiomyopathy panel; DCM/LVNC, dilated cardiomyopathy/left ventricular non-compaction panel; HCM, hypertrophic cardiomyopathy panel; MFLS, Marfan syndrome/Loeys–Dietz syndrome panel; TAAD, thoracic aortic aneurysms and dissections panel.

**Figure 3:**
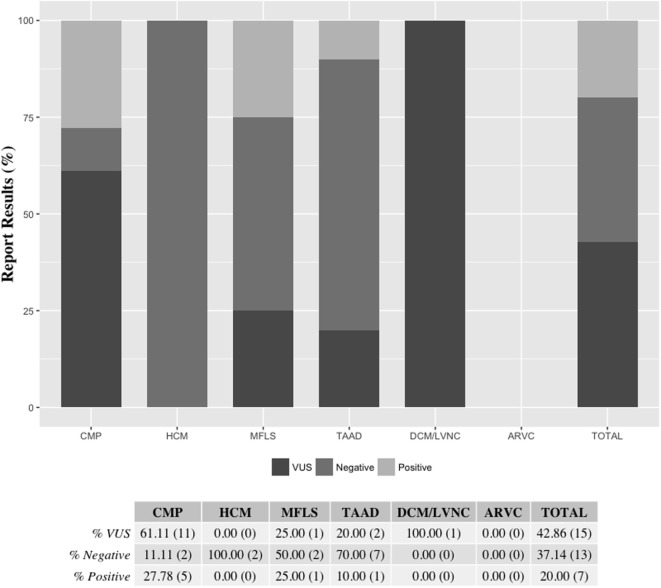
**Reported results per panel requested**. Final patient panel results were reported as either being positive (at least one pathogenic or likely pathogenic variant was found), VUS (at least one VUS but no pathogenic or likely pathogenic variant was found), or negative (no pathogenic, likely pathogenic, or VUS was found). The ARVC panel was not ordered for patient testing during the time-frame selected. Two patients (IDs 24 and 32) had sequencing reported for two panels (CMP as a reflex) Percentages are shown followed by the actual number of reports of each category in parenthesis. ARVC, arrhythmogenic right ventricular cardiomyopathy panel; CMP, comprehensive cardiomyopathy panel; DCM/LVNC, dilated cardiomyopathy/left ventricular non-compaction panel; HCM, hypertrophic cardiomyopathy panel; MFLS, Marfan syndrome/Loeys–Dietz syndrome panel; TAAD, thoracic aortic aneurysms and dissections panel; VUS, variant of uncertain clinical significance.

Results from de-identified patient samples received by our clinical laboratory in the period of January 2015 to December 2015 with requisition for testing using one the cardiovascular NGS panels were collected. The patient reported results and variants found per panel requested are detailed in Table S8 in Supplementary Material. Overall, we were requested to perform NGS tests for 33 patients in the period selected for the writing of this manuscript, with two patients (IDs 24 and 32) having sequencing reported for two panels (CMP as a reflex). The distribution of the type of panel requested reflects the specific patient population of the requesting health professional, for which they deem to have the necessity to order a clinical genetic testing. Out of the 35 panels requested for testing, about half (18) were CMP panels. The second most ordered NGS test was the TAAD panel (10, or 29%). There were no requests for the ARVC panel during the period selected (Figure 2). Overall, 20% of all tests requested resulted in a positive result, meaning that at least one pathogenic or likely pathogenic variant was found in the patient tested. The CMP panel had the highest positive result rate with 28% of patients tested being reported to have at least one variant that could explain their phenotype. Additionally, about 43% of all NGS tests were reported to have no pathogenic or likely pathogenic variants, but to have at least one VUS, and about 37% of panels tested had a negative result (no pathogenic, likely pathogenic, or VUS was found). All HCM panels were reported as negative; however, only two HCM panels were requested in the period analyzed (Figure 3). Figure 3 shows a graphic representation of our clinical sample population pick-up rates. Overall, the positive rate of approximately 28% obtained for the CMP panel (our largest sample size) is consistent with the previously published expected positive rate of 30% for patients diagnosed with DCM—we compare the published DCM rate to our CMP rate since in most cases, when the ordering physicians for our patients suspected a diagnosis of DCM they tended to order the larger, more comprehensive CMP panel (28). For other panels, the positive rates were heavily dependent on the diagnostic criteria and interpretation of clinical presentation used by the clinician prior to patient genetic testing. Additionally, in many cases, the panel requested by the physician may have been used as a differential diagnosis to exclude a specific disease. Therefore, the rate we obtained for the HCM, MFLS, TAAD, and DCM/LVNC panels may not be an accurate representation of expected pick-up rates for a given definitively diagnosed patient population.

## Discussion

Sequencing information may be used as an aid to clinicians in determining disease diagnosis, follow-up procedures, genetic counseling, therapeutic strategy, and treatment of disorders based on variants found in the gene(s) analyzed. Laboratory-developed NGS panel tests can identify an individual’s genotype from genomic DNA with focus on specific disorders, groups of genes, phenotypes, and other variables in an efficient and cost-effective way. In this study, we present our results from the optimization and validation of the Illumina TSO NGS panel utilizing the Illumina MiSeq and an in-house bioinformatics pipeline for clinical testing of cardiovascular disorders (including HCM, DCM/LVNC, ARVC, MFS/LDS, TAAD, and comprehensive CMP). Our validation demonstrated that our procedures fulfilled the requirements of a clinical assay for detection of nucleotide base alterations, and small deletions and insertions with a desirable clinical test level of quality to detect constitutive genomic variants. Compared to the use of Sanger sequencing, at the current pricing and established turnaround time for clinical samples at our laboratory, one would save approximately 9.5 times the cost, and 10.2 times the time when using our NGS approach for an average gene, such as *LMNA*. Compared to other NGS targeting technologies, the hybridization capture-based approach that we used (as opposed to amplicon-based approach) allowed us to obtain a high quality NGS panel with clinically acceptable sensitivity, specificity, accuracy, precision, and coverage. Previous studies have shown that amplicon methods tend to be suboptimal and may generate higher FP and FN rates as well as lower coverage and uniformity (29). Finally, with regards to our optimized bioinformatics pipeline, we employed the most widely used tools to identify variants following the best practices of the GATK. Although, to our knowledge, there is no single state-of-the-art pipeline that is currently available for clinical NGS panel studies, our validation studies of our in-house developed bioinformatics pipeline have also shown clinically acceptable high quality results. A limitation of our study is that it is based on a small sample size, which may render it to be of insufficient power to address the genotypic variability of future samples and the true analytic sensitivity and specificity. Future studies are necessary to increase the power of our current assessment.

Our sub-panel approach included the selection of genes associated with cardiovascular diseases according to disease phenotype. Among the genes selected for each panel, several belong to a list of known pathogenic (KP) and/or expected pathogenic (EP) actionable variants, according to the ACMG recommendations on incidental findings: 18 genes with actionable KP/EP variants out of 61 CMP panel genes (*MYBPC3, MYH7, TNNT2, TNNI3, TPM1, MYL3, ACTC1, PRKAG2, GLA, MYL2, LMNA, RYR2, PKP2, DSP, DSC2, TMEM43, DSG2*, and *SCN5A*), 10/18 HCM panel genes (*MYBPC3, MYH7, TNNT2, TNNI3, TPM1, MYL3, ACTC1, PRKAG2, GLA*, and *MYL2*), 8/33 DCM/LVNC panel genes (*MYBPC3, MYH7, TNNT2, TNNI3, TPM1, ACTC1, LMNA*, and *SCN5A*), 6/8 ARVC panel genes (*LMNA, PKP2, DSP, DSC2, TMEM43*, and *DSG2*), 3/3 MFS/LDS panel genes (*FBN1, TGFBR1*, and *TGFBR2*), and 8/18 TAAD panel genes (*COL3A1, FBN1, TGFBR1, TGFBR2, SMAD3, ACTA2, MYLK*, and *MYH11*) (30).

In our experience with 33 patients referred for clinical genetic testing using the given NGS panels, we found a positive result (pathogenic or likely pathogenic variant) for 20% of the panels tested. The highest positive rate resulted from CMP panels (28%), which was also the NGS panel that was the most requested in the period analyzed (51% of all panels requested). Patients with a positive test result may have a more appropriate management of their clinical phenotype and they were, as well as their relatives, recommended to receive continued clinical evaluation, follow-up, and genetic counseling. Our laboratory offers targeted testing for the specific variant(s) detected in the proband to at-risk relatives using Sanger sequencing technology, and many of the families took advantage of this service.

From the 33 patients referred for clinical genetic testing using the given NGS panels, about 43% of all tests were reported to have at least one VUS, but not a definitive pathogenic or likely pathogenic variant. The functional significance of these variants is not known at present and their contribution to the patient’s disease phenotype could not be determined at the time of reporting. However, these VUSs are good candidates for functional studies, and the analysis of other affected relatives of the patient tested may help support a potential pathogenic role of these variants if they co-segregate with the disease phenotype in the families studied.

From the 33 patients referred for clinical genetic testing using the given NGS panels, about 37% of all tests were reported to be negative. Many reasons may be related to a negative result. For example, a complicated clinical phenotype, or confounding factors such as environmental causes may result in a challenging choice for the most appropriate test to run in order to achieve the diagnosis of the proband. On the laboratory side, there are several technical limitations that could be associated with negative results. For example, the enrichment design employed in the commercial kit used for our NGS assays targets and detects variants in the coding sequence and adjacent splicing and intronic sequences of the desired genes, while variants in deep intronic, non-coding, and regulatory regions that could affect gene expression were not targeted by our NGS assays. In addition, our clinical NGS tests were not designed for the purpose of detecting copy number variants due to large deletions and duplications encompassing all or a large portion of a gene (the maximum length of indels we detected was of 23 nucleotides in a homozygous state and 31 nucleotides in a heterozygous state). Moreover, our NGS methodology and depth of coverage were designed for constitutional genetics and may not detect low level mosaicism. Likewise, there could be some coding and splice site regions of genes that may present with an intrinsic sequence characteristics leading to suboptimal data. Finally, although our panels have been designed to include the great majority of genes known to be involved in each of the cardiovascular disorders listed here, every day, scientific progress reveals new genes that may be causing or be associated with these diseases. A benefit of our sub-panel design approach, in which a large panel was subdivided into smaller panels, is the fact that new genes and new sub-panels may be quickly validated from the list of 4,813 genes in the TSO panel as new literature points to new genes being involved in cardiovascular diseases. This validation would only consist of developing Sanger sequencing for regions of systematic low coverage and regions of known SegDups for the new genes and the calculation of parameters, such as FN, FP, accuracy, sensitivity, and specificity. For example, we are currently working on the validation and clinical implementation of NGS sub-panels for testing in Noonan spectrum disorders, long QT syndrome, hypertension, lipid disorders, and comprehensive arrhythmias. Additionally, our TSO panel and validation strategy may be used in the future for an array of non-cardiovascular diseases, including neurological, metabolic, skeletal disorders, and cancer, to name a few.

## Websites

1000 genomes browser (URL: http://www.ncbi.nlm.nih.gov/variation/tools/1000genomes/).
1000 G project release (genotypic information for NA12003, NA19449, NA19982, NA19704, NA11931, NA11829, and NA06986) (URL: ftp://ftp-trace.ncbi.nih.gov/1000genomes/ftp/release/20110521/).
Bioinformatics pipeline scripts (http://compbio.iupui.edu/group/6/pages/clinicalsequencing).
Coriell Cell Repositories, Camden, NJ, USA (URL: http://ccr.coriell.org/).
dbSNP (URL: http://www.ncbi.nlm.nih.gov/SNP/).
GATK UnifiedGenotyper (URL: https://www.broadinstitute.org/gatk/gatkdocs/org_broadinstitute_gatk_tools_walkers_genotyper_UnifiedGenotyper.php#–heterozygosity).
Illumina (URL: http://www.illumina.com/).
NA12878 genotype information (URL: https://www.illumina.com/platinumgenomes.html).
NA19240 genotype information (URL: ftp://ftp-trace.ncbi.nih.gov/1000genomes/ftp/pilot_data/release/2010_07/trio/snps/).
Picard-tools-1.105 (URL: http://picard.sourceforge.net).
TSO Data Sheet (URL: https://www.illumina.com/content/dam/illumina-marketing/documents/products/datasheets/datasheet_trusight_one_panel.pdf).
TSO Full Gene List (URL: https://www.illumina.com/products/by-type/clinical-research-products/trusight-one.html).
TSO Technical Note (URL: https://www.illumina.com/content/dam/illumina-marketing/documents/products/technotes/technote_trusight_one_panel.pdf).
UCSC browser (URL: http://genome.ucsc.edu/).

## Ethics Statement

The study is exempt from the Helsinki declaration for studies involving human subjects because it either employed commercial human cell lines (from Coriell Institute) or de-identified and anonymized human subjects specimens.

## Author Contributions

PC-S, HG, YL, and MV were responsible for conception and design of the experiments and manuscript drafting. PC-S, HG, TL, and HL performed the experiments. PC-S, HG, TL, HL, YL, and MV were responsible for data generation, analysis, and interpretation. PC-S, HG, TL, HL, YL, PS-C, and MV were responsible for revising the content critically for intellectual content and for final approval of the manuscript.

## Conflict of Interest Statement

PC-S, TL, and MV are members of the Indiana University Molecular Diagnostic Laboratory.
