# Supplementary material for: Validation and Utilization of a Clinical Next-Generation Sequencing Panel for Selected Cardiovascular Disorders
Source: Front Cardiovasc Med. 2017 Mar 15;4:11. doi: 10.3389/fcvm.2017.00011 (PMC5350117; doi:10.3389/fcvm.2017.00011)
Supplement: Supplementary file 2 [file Data_Sheet_1.DOCX]

**Validation and utilization of a clinical next generation sequencing panel for selected cardiovascular disorders**

Patrícia B. S. Celestino-Soper^1^, Hongyu Gao^1^, Ty C. Lynnes^1^, Hai Lin^1,2^, Yunlong Liu^1,2^, Katherine G. Spoonamore^3^, Peng-Sheng Chen^3^, Matteo Vatta^1,3*^

1 Department of Medical and Molecular Genetics, Indiana University School of Medicine, Indianapolis, IN, USA 2 Center for Computational Biology and Bioinformatics, Indiana University Purdue University Indianapolis, Indianapolis, IN, USA 3 Krannert Institute of Cardiology, Division of Cardiology, Department of Medicine, Indiana University School of Medicine, Indianapolis, IN, USA.

**Correspondence:**

Dr. Matteo Vatta

Indiana University School of Medicine

Department of Medical and Molecular Genetics

550 University Blvd, UH AOC 6029

Indianapolis, IN 46202

mvatta@iu.edu

Keywords: next generation sequencing, sequencing panels, cardiovascular, panel validation, clinical sequencing.

**Supplementary Materials and methods**

**Bioinformatics pipeline**

Bioinformatics validation/revalidation pipeline

The bioinformatics pipeline was validated on various samples sequenced as described in Supplementary Table 2. Analytical targets included the TruSight One panel targeting 4,813 clinically relevant genes composed of 62,309 regions and a HaloPlex cardiovascular gene panel that was custom designed to resolve 246 target genes comprising 4,227 regions [1]. Information from the latter was used for the purpose of confirming variants in samples run in both panels.

The bioinformatics pipeline described was used to analyze the resulting MiSeq 150-bp pair-end sequence data. In order to assess each NGS run performance, alignment summary metrics to report alignment quality (including percentage of reads mapped to the reference) and metrics specific to target capture, analyses were generated using picard-tools-1.105 (evaluated the performance of target enrichment protocol, listed numbers of sequenced and aligned bases, as well as bases on, near, and off-bait). The percent coverage and minimum and maximum sequence depth for each target region were reported in the depth of coverage metrics. In-house scripts (available at http://compbio.iupui.edu/group/6/pages/clinicalsequencing) were written to access/validate the performance of sequencing and the bioinformatics pipeline, including sensitivity, specificity, and precision (intra-run repeatability and inter-run reproducibility). The variants of the HapMap samples identified by the pipeline were compared with the 1000 Genomes variant calls of the corresponding samples.

Considering allelic fraction and zygosity, the allele zygosity (homozygous or heterozygous variants) was determined by the GATK UnifiedGenotyper. Supplementary Table 4 (GT (genotype) field/column) shows the GT encoded as allele values separated by either / or |. The allele value 0 is for reference allele (REF field), 1 is for the first alternative allele (ALT field), 2 is for the second alternative allele and so on. Therefore, 0/1 is a heterozygous call whereas 1/1 is a homozygous call. The read depth for each allele is in the AD column. Allelic depth for the reference and alternative alleles are in the order listed. The confidence of a genotype is defined using the Phred-scaled likelihood (PL column) and it shows three genotype likelihoods in the order of AA (homozygous reference allele), AB (heterozygous), BB (homozygous alternative allele). The higher the PL value, the less likely is being that genotype.

For the determination of the accuracy, precision (reproducibility), analytic sensitivity (ability to detect a mutation when the mutation is present; calculated as 1-false negative rate), and analytic specificity (ability to give a negative result when the specimen does not have a mutation; calculated as 1-false positive rate) for all variant types to be assessed by the test (e.g. single nucleotide variants, indels, structural variants, homopolymers, copy number variants), the following metrics were used to measure the analytic performance:

*Variant evaluation metrics:* includes the number and percentage of true positive (TP), false negative (FN; calculated as 1-sensitivity), false positive (FN; calculated as 1-specitivity), variant call precision and recall rate, analytic sensitivity and analytic specificity. Analytic sensitivity and specificity for indels were not assessed due to the insufficient number of indels found for the samples tested (2 indels from 34 samples tested for TAAD panel, 13 from 34 samples for HCM panel, 117 from 34 samples for CMP panel, 1 from 34 samples for MFS/LDS panel, 65 from 34 samples for DCM/LVNC panel, 13 from 34 samples for ARVC panel), which would result in insufficient power for statistical analyses.

*Precision metrics including intra-run repeatability and inter-run reproducibility:* non-reference sensitivity (NRS), Non-reference discrepancy (NRD) and genotype concordance were calculated as previously published [2, 3].

For indels, the maximum length properly detected by the TSO panel (based on genotype information for the NA12878 sample from the Illumina Platinum Genome project) was of 22 nucleotides in a homozygous state (rs111033223) and 30 nucleotides in a heterozygous state (rs199508964) (both cases with satisfactory quality and sequence depth).

Additionally, our bioinformatics pipeline identified and flagged variants in target regions that have segmental duplication or highly homologous sequences (the segment duplication database hg19_genomicSuperDups.txt was downloaded with ANNOVAR). Any variants found within these regions were to be individually assessed for need and possibilities of confirmation by orthogonal procedures, such as Sanger (or Big Dye) sequencing.

NGS bioinformatics pipeline - quality management (QM) program, updates, data storage, version traceability, exception log, data transfer and confidentiality policy

The laboratory followed a series of procedures for the QM program, updates, data storage, version traceability, exception log, data transfer and confidentiality policy for all NGS clinical panels, following the College of American Pathologists (CAP) guidelines [4]. NGS data received and generated from running the bioinformatics pipeline were stored in an in-house server that is Health Insurance Portability and Accountability Act (HIPAA) compliant. The raw sequence imaging data will be stored for at least two years in a HIPAA compliant server. The original raw sequence data (FASTQ format), the alignment file (BAM format), the variant file (VCF format), and the final report file which includes ANNOVAR and HGMD annotation together with variant information from the variant file will be kept on the server for a minimum of 20 years. Regarding version traceability, a log file was generated for each analysis run. Reference genome build, database versions and any other in-house scripts applied were recorded for each subject.

Sequence variants – output

An output file was generated as described in the bioinformatics pipeline section. The variant report utilized the HUGO Gene Nomenclature Committee (HGNC) gene names and the REFseq accession numbers. Interpretation and reporting was performed as described in the “Post-bioinformatics analyses” section. A flowchart of the complete bioinformatics pipeline is depicted in Supplementary Figure 1.

**Post-bioinformatics analyses**

Variants present in HGMD database - pathogenic/likely pathogenic if:

1. The variant (truncating, splicing, missense, synonymous, etc.) was identified in a gene with extensive linkage data in a large family or in multiple families with co‐segregation with disease in affected subjects from a multigenerational family or in affected from multiple families, **or…**
2. The variant appears *de novo* in the patient in the literature (paternity and maternity confirmed) **and** the variant has well‐established functional studies showing a deleterious effect, **or…**
3. The variant affects the same amino acid change as an established pathogenic variant, **or…**
4. Variant identified sporadically (without parents’ study) is truncating in a gene where loss of function (LOF) is an established mechanism of disease, **or…**
5. Variant with prevalence in affected individuals is statistically increased over controls in very large and replicated studies with reported functional deleterious effect, **or…**
6. HGMD variants with deleterious functional effect **and** the variant has frequency <1% in the exome sequencing project (ESP) database **and** 1000Genomes database **or** ≥ 1% but disproportionate frequency in specific ethic groups (>5X difference due to founder effect), **or…**
7. HGMD variant may or may not be sufficient in isolation or is a very strong susceptibility variant (such as D85N in *KCNE1*). These variants may, for example, have disease association and have functional studies showing the variant is deleterious and have frequency < 5% in ESP database **and** 1000Genomes database (or frequency ≤ 5% but disproportionate frequency in specific ethic groups, i.e., >5X difference due to founder effect).
8. For all variants reported in HGMD that are classified as **splicing** (**+/-** 2 bp from exon), if no functional data is presented in the literature, a frequency < 1% in ESP database **and** 1000Genomes database **or** frequency ≥ 1% but disproportionate in specific ethic groups (>5X difference due to founder effect) will determine that the splicing variant is **pathogenic/likely pathogenic**. **Otherwise**, the variant is benign/likely benign.
9. If the HGMD variant is exonic **synonymous** or exonic **nonsynonymous**, **and** the variant is located in the 1^st^ nucleotide of an exon **or** the last two nucleotides of an exon (use coordinates to check position in UCSC browser for any known isoform), **and** the variant has a frequency < 1% in ESP database **and** 1000Genomes database **or** the variant has frequency ≥1% but disproportionate in specific ethic groups (> 5X difference due to founder effect), **then**, …

i.1) if the variant results in ≥ 33% reduction in splicing score compared to the reference sequence (using the following freely available prediction softwares), **and** was previously reported as deleterious in other databases (ClinVar/dbSNP, Pubmed, Google, etc) = **pathogenic/likely pathogenic**.

<http://www.fruitfly.org/seq_tools/splice.html> (preferred)

<http://www.umd.be/HSF/>

<http://www.cbs.dtu.dk/services/NetGene2/>

**NOTE:** We used 33% because, although the score is by no means a measure of the effect size, it represents a sufficient threshold to identify potentially functional active variants. The splice score alone is not used and cannot be used as the sole evidence for pathogenicity, but a score drop larger than 33% represents evidence that may be used.

**NOTE:** if first software (fruit fly) does not show ≥ 33% reduction in splicing score, STOP (don’t test the other 2 softwares). If the first software (fruit fly) shows ≥ 33% reduction in splicing score, then the other 2 softwares must also show some degree of reduction (not necessarily 33%) to be considered sufficient evidence.

**NOTE:** if the change is not in the first or last codon and the frequency is high, the variant is benign and no software prediction is necessary.

i.2) If there is no functional data is presented in the literature **and** the variant results in ≥ 33% reduction in splicing score (using the prediction softwares above), **and** the variant was not previously reported in other databases ClinVar/dbSNP, Pubmed, Google, etc) = **VUS.**

i.3) If there is no published functional data and the variant results in < 33% reduction in splicing score (using the prediction softwares above) = **benign/likely benign**.

1. If the HGMD variant is classified by the bioinformatics pipeline as “intronic”, follow the same rational as in step I (as applicable) to determine its classification.

**NOTE:** *TTN* HGMD variants must have functional studies for any of the options so the variant will be classified as pathogenic/likely pathogenic. If no functional studies in a *TTN* variant, it will be classifies as VUS.

Variants present in HGMD database - gene modifier if:

Variant with deleterious functional effect but with frequency ≥ 1% in ESP database **or** 1000Genomes database, **and** there is no disproportionate frequency in specific ethic groups (>5X difference due to founder effect), **and** clinical phenotype of reported literature case is similar to the phenotype of laboratory patient under analysis. **Otherwise**, the variant is benign/likely benign.

Variants present in HGMD database - variant of unknown significance (VUS), or undetermined if:

a. The variant has some evidence of potential pathogenicity (identified in patients but not in controls in the literature), **and** the variant has deleterious prediction from literature (but not enough evidence of pathogenicity), **and** the variant frequency < 1% in ESP database **and** 1000Genomes database **or** variant frequency ≥1% but there is disproportionate frequency in specific ethic groups (>5X difference due to founder effect), **or …**

- 1. The variant is reported in the literature to have disease association (but no functional data available), **and** the variant is reported to be deleterious in the majority of online prediction databases (PolyPhen-2, Sift, Mutation Taster, SNP&Go), **and** the variant frequency <1% in ESP database **and** 1000Genomes database **or** variant frequency ≥1% but there is disproportionate frequency in specific ethic groups (>5X difference due to founder effect), **or …**
  2. The variant is reported in the literature to have deleterious prediction (but no disease association available), **and** the variant frequency <1% in ESP database **and** 1000Genomes database **or** variant frequency ≥1% but there is disproportionate frequency in specific ethic groups (>5X difference due to founder effect).

Variants present in HGMD database - benign/likely benign if:

a. Steps above do not apply (for example, high frequency in controls), or the variant is a weak modifier and there is evidence that it is tolerated, **or …**

b. There are functional data showing that the effect of the variant is similar to WT.

Variants not present in HGMD database (non_HGMD) - intronic, intergenic or UTRs:

Not reported.

Variants not present in HGMD database (non_HGMD) – splicing:

a. If the variant has strong genetic/functional data **and** was previously reported as deleterious in other databases (ClinVar/dbSNP, Pubmed, Google, etc) = **pathogenic/likely pathogenic.**

b. If the variant is in a gene where loss of function (LOF) or haploinsufficiency is an established mechanism of disease (for example, *LMNA*) = **pathogenic/likely pathogenic** (independent of frequency in ESP database **and** 1000Genomes database **-** unless the human reference is the one with a variant).

c. If the variant is not previously reported in other databases ClinVar/dbSNP, Pubmed, Google, etc), **and** is in a gene where loss of function (LOF) or haploinsufficiency is **NOT** an established mechanism of disease = **VUS** (independent of frequency in ESP database **and** 1000Genomes database **-** unless the human reference is the one with a variant).

d. If the variant is found to be *de novo* in the given patient, **and** there is strong genetic/functional data for the variant, **and** the variant has a frequency < 1% in ESP database **and** 1000Genomes database **or** the variant has frequency ≥1% but disproportionate in specific ethic groups (> 5X difference due to founder effect) = **pathogenic/likely pathogenic.**

e. If the variant has a frequency <1% in ESP database **and** 1000Genomes database **or** the variant has frequency ≥1% but disproportionate in specific ethic groups (> 5X difference due to founder effect) = **pathogenic/likely pathogenic.**

f. If the variant has a frequency > 1% in ESP database **or** 1000Genomes database **without** being disproportionate in specific ethic groups (> 5X difference due to founder effect) = **benign/likely benign.**

g. If the variant is a weak modifier and there is evidence that it is tolerated = **benign/likely benign.**

h. If the variant is categorized as benign or likely benign in other databases (ClinVar/dbSNP, Pubmed, Google, etc) with supporting evidence (for example, that the effect of the variant is similar to WT) = **benign/likely benign.**

i. If the variant is recurrent in the Molecular Genetics Laboratory (MGL) in-house database of subjects with different underlying diagnosis = **benign/likely benign.**

**NOTE:** For *TTN* splicing variants at the A-band will be classified as likely pathogenic, while at the other positions would be classified as VUS.

Variants not present in HGMD database (non_HGMD) – stop gain:

a. If the variant has strong genetic/functional data **and** was previously reported as deleterious in other databases (ClinVar/dbSNP, Pubmed, Google, etc) = **pathogenic/likely pathogenic.**

b. If the variant is in a gene where loss of function (LOF) or haploinsufficiency is an established mechanism of disease (for example, *LMNA*) = **pathogenic/likely pathogenic** (independent of frequency in ESP database **and** 1000Genomes database **-** unless the human reference is the one with a variant).

c. If the variant is not previously reported in other databases ClinVar/dbSNP, Pubmed, Google, etc), **and** is in a gene where loss of function (LOF) or haploinsufficiency is **NOT** an established mechanism of disease = **VUS** (independent of frequency in ESP database **and** 1000Genomes database - unless the human reference is the one with a variant).

d. If the variant is found to be *de novo* in the given patient, **and** there is strong genetic/functional data for the variant, **and** the variant has a frequency < 1% in ESP database **and** 1000Genomes database **or** the variant has frequency ≥1% but disproportionate in specific ethic groups (> 5X difference due to founder effect) = **pathogenic/likely pathogenic.**

e. If the variant has a frequency < 1% in ESP database **and** 1000Genomes database **or** the variant has frequency ≥1% but disproportionate in specific ethic groups (> 5X difference due to founder effect) = **pathogenic/likely pathogenic.**

f. If the variant has a frequency > 1% in ESP database **or** 1000Genomes database **without** being disproportionate in specific ethic groups (> 5X difference due to founder effect) = **benign/likely benign.**

g. If the variant is a weak modifier and there is evidence that it is tolerated = **benign/likely benign.**

h. If the variant is categorized as benign or likely benign in other databases (CLinVar/dbSNP, Pubmed, Google, etc) with supporting evidence (for example, that the effect of the variant is similar to WT) = **benign/likely benign.**

i. If the variant is recurrent in the MGL in-house database of subjects with different underlying diagnosis = **benign/likely benign.**

**NOTE:** For *TTN* stop gain variants at the A-band will be classified as likely pathogenic, while at the other positions would be classified as VUS.

Variants not present in HGMD database (non_HGMD) – frameshift indels:

a. If the variant has strong genetic/functional data **and** was previously reported as deleterious in other databases (ClinVar/dbSNP, Pubmed, Google, etc) = **pathogenic/likely pathogenic.**

b. If the variant is in a gene where loss of function (LOF) or haploinsufficiency is an established mechanism of disease (for example, *LMNA*) = **pathogenic/likely pathogenic** (independent of frequency in ESP database **and** 1000Genomes database - unless the human reference is the one with a variant).

c. If the variant is not previously reported in other databases ClinVar/dbSNP, Pubmed, Google, etc), **and** is in a gene where loss of function (LOF) or haploinsufficiency is **NOT** an established mechanism of disease = **VUS** (independent of frequency in ESP database **and** 1000Genomes database **-** unless the human reference is the one with a variant).

d. If the variant is found to be *de novo* in the given patient, **and** there is strong genetic/functional data for the variant, **and** the variant has a frequency <1% in ESP database **and** 1000Genomes database **or** the variant has frequency ≥1% but disproportionate in specific ethic groups (> 5X difference due to founder effect) = **pathogenic/likely pathogenic.**

e. If the variant has a frequency <1% in ESP database **and** 1000Genomes database **or** the variant has frequency ≥1% but disproportionate in specific ethic groups (>5X difference due to founder effect) = **pathogenic/likely pathogenic.**

f. If the variant has a frequency > 1% in ESP database **or** 1000Genomes database **without** being disproportionate in specific ethic groups (> 5X difference due to founder effect) = **benign/likely benign.**

g. If the variant is a weak modifier and there is evidence that it is tolerated = **benign/likely benign.**

h. If the variant is categorized as benign or likely benign in other databases (CLinVar/dbSNP, Pubmed, Google, etc) with supporting evidence (for example, that the effect of the variant is similar to WT) = **benign/likely benign.**

i. If the variant is recurrent in the MGL in-house database of subjects with different underlying diagnosis = **benign/likely benign.**

**NOTE:** For *TTN* frameshift variants at the A-band will be classified as likely pathogenic, while at the other positions would be classified as VUS.

Variants not present in HGMD database (non_HGMD) – non-frameshift indels:

a. If the variant has a frequency <1% in ESP database **and** 1000Genomes database **or** the variant has frequency ≥1% but disproportionate in specific ethic groups (> 5X difference due to founder effect), **and** the variant has strong genetic/functional data **and** was previously reported as deleterious in other databases (ClinVar/dbSNP, Pubmed, Google, etc) = **pathogenic/likely pathogenic.**

b. If the variant has a frequency <1%in ESP database **and** 1000Genomes database **or** the variant has frequency ≥1% but disproportionate in specific ethic groups (> 5X difference due to founder effect), **and** the variant was not previously reported in other databases ClinVar/dbSNP, Pubmed, Google, etc) = **VUS.**

c. If the variant has a frequency > 1% in ESP database **or** 1000Genomes database **without** being disproportionate in specific ethic groups (> 5X difference due to founder effect) = **benign/likely benign.**

d. If the variant is not inserting/deleting amino acids in expanded (repetitive) regions (for example, Poly Q or poly A stretches), **and** has a frequency <1% in ESP database **and** 1000Genomes database **or** frequency ≥1% but disproportionate in specific ethic groups (>5X difference due to founder effect), **and** the variant was not previously reported in other databases ClinVar/dbSNP, Pubmed, Google, etc) = **VUS.**

e. If the variant is not inserting/deleting amino acids in expanded (repetitive) regions (for example, Poly Q or poly A stretches), **and** has a frequency > 1% in ESP database **and** 1000Genomes database **without** being disproportionate in specific ethic groups (> 5X difference due to founder effect) = **benign/likely benign.**

f. If the variant is inserting/deleting amino acids in expanded (repetitive) regions (for example, Poly Q or poly A stretches), **and** has a frequency <1% in ESP database **and** 1000Genomes database **or** frequency ≥1% but disproportionate in specific ethic groups (>5X difference due to founder effect) = **VUS.**

g. If the variant is a weak modifier and there is evidence that it is tolerated = **benign/likely benign.**

h. If the variant is categorized as benign or likely benign in other databases (CLinVar/dbSNP, Pubmed, Google, etc) with supporting evidence (for example, that the effect of the variant is similar to WT) = **benign/likely benign.**

i. If the variant is recurrent in the MGL in-house database of subjects with different underlying diagnosis = **benign/likely benign.**

**NOTE:** For *TTN* non-frameshift variants will be classified as benign/likely benign.

Variants not present in HGMD database (non_HGMD) – stop loss:

a. If the variant has a frequency <1% in ESP database **and** 1000Genomes database **or** the variant has frequency ≥1% but disproportionate in specific ethic groups (> 5X difference due to founder effect), **and** the variant has strong genetic/functional data **and** was previously reported as deleterious in other databases (ClinVar/dbSNP, Pubmed, Google, etc) = **pathogenic/likely pathogenic.**

b. If the variant has a frequency <1%in ESP database **and** 1000Genomes database **or** the variant has frequency ≥1% but disproportionate in specific ethic groups (> 5X difference due to founder effect), **and** the variant was not previously reported in other databases ClinVar/dbSNP, Pubmed, Google, etc) = **VUS.**

c. If the variant has a frequency > 1% in ESP database **or** 1000Genomes database **without** being disproportionate in specific ethic groups (> 5X difference due to founder effect) = **benign/likely benign.**

d. If the variant is a weak modifier and there is evidence that it is tolerated = **benign/likely benign.**

e. If the variant is categorized as benign or likely benign in other databases (CLinVar/dbSNP, Pubmed, Google, etc) with supporting evidence (for example, that the effect of the variant is similar to WT) = **benign/likely benign.**

f. If the variant is recurrent in the MGL in-house database of subjects with different underlying diagnosis = **benign/likely benign.**

**NOTE:** For *TTN* stop loss variants will be classified as benign/likely benign.

Variants not present in HGMD database (non_HGMD) – nonsynonymous (missense):

a. If the variant has a frequency <1% in ESP database **and** 1000Genomes database **or** the variant has frequency v but disproportionate in specific ethic groups (> 5X difference due to founder effect), **and** the variant has strong genetic/functional data **and** was previously reported as deleterious in other databases (ClinVar/dbSNP, Pubmed, Google, etc) = **pathogenic/likely pathogenic** (independent of online prediction databases).

b. If the variant has is reported to be deleterious in the majority of online prediction databases (PolyPhen-2, Sift, Mutation Taster, SNP&Go), **and** has a frequency <1%in ESP database **and** 1000Genomes database **or** frequency ≥1% but disproportionate in specific ethic groups (> 5X difference due to founder effect), **and** the variant was not previously reported in other databases ClinVar/dbSNP, Pubmed, Google, etc) = **VUS.**

c. If the variant has a frequency > 1% in ESP database **or** 1000Genomes database **without** being disproportionate in specific ethic groups (> 5X difference due to founder effect) = **benign/likely benign.**

d. If the variant was not previously reported in other databases ClinVar/dbSNP, Pubmed, Google, etc), **and** it is reported to be deleterious in the majority of online prediction databases (PolyPhen-2, Sift, Mutation Taster, SNP&Go) = **benign/likely benign** (independent of the frequency in ESP database **and** 1000Genomes database).

e. If the variant is a weak modifier and there is evidence that it is tolerated = **benign/likely benign.**

f. If the variant is categorized as benign or likely benign in other databases (CLinVar/dbSNP, Pubmed, Google, etc) with supporting evidence (for example, that the effect of the variant is similar to WT) = **benign/likely benign.**

g. If the variant is recurrent in the MGL in-house database of subjects with different underlying diagnosis = **benign/likely benign.**

**NOTE:** For *TTN* nonsynonymous variants will be classified as benign/likely benign.

Variants not present in HGMD database (non_HGMD) – synonymous (silent):

a. If the variant has a frequency <1% in ESP database **and** 1000Genomes database **or** the variant has frequency ≥1% but disproportionate in specific ethic groups (> 5X difference due to founder effect), **and** the variant has strong genetic/functional data **and** was previously reported as deleterious in other databases (ClinVar/dbSNP, Pubmed, Google, etc) = **pathogenic/likely pathogenic**.

b. If the variant has a frequency <1% in ESP database **and** 1000Genomes database **or** the variant has frequency ≥1% but disproportionate in specific ethic groups (> 5X difference due to founder effect), **and** the variant is located in the 1^st^ nucleotide of an exon **or** the last two nucleotides of an exon (see figure “Nucleotides of interest within first and last codons of exon”), **and** (there is no published functional data) the variant results in ≥ 33% reduction in splicing score compared to the reference sequence (using the following prediction softwares), **and** was previously reported as deleterious in other databases (ClinVar/dbSNP, Pubmed, Google, etc) = **pathogenic/likely pathogenic**.

<http://www.fruitfly.org/seq_tools/splice.html> (preferred)

<http://www.umd.be/HSF/>

<http://www.cbs.dtu.dk/services/NetGene2/>

**NOTE:** If first software (fruit fly) does not show ≥ 33% reduction in splicing score, STOP (don’t test the other 2 softwares). If the first software (fruit fly) shows ≥ 33% reduction in splicing score, then the other 2 softwares must also show some degree of reduction (not necessarily 33%) to be considered sufficient evidence.

c. If the variant has a frequency <1% in ESP database **and** 1000Genomes database **or** the variant has frequency ≥1% but disproportionate in specific ethic groups (>5X difference due to founder effect), **and** the variant is located in the 1^st^ nucleotide of an exon **or** the last two nucleotides of an exon (see figure below), **and** (there is no published functional data) the variant results in ≥ 33% reduction in splicing score (using the prediction softwares above), **and** the variant was not previously reported in other databases ClinVar/dbSNP, Pubmed, Google, etc) = **VUS.**

d. If the variant has a frequency <1% in ESP database **and** 1000Genomes database **or** the variant has frequency ≥1% but disproportionate in specific ethic groups (> 5X difference due to founder effect), **and** the variant is located in the 1^st^ nucleotide of an exon **or** the last two nucleotides of an exon (see figure below), **and** (there is no published functional data) the variant results in < 33% reduction in splicing score (using the prediction softwares above) = **benign/likely benign**.

e. If the variant has a frequency > 1% in ESP database **or** 1000Genomes database **without** being disproportionate in specific ethic groups (>5X difference due to founder effect) = **benign/likely benign.**

f. If the variant is a weak modifier and there is evidence that it is tolerated = **benign/likely benign.**

g. If the variant is categorized as benign or likely benign in other databases (CLinVar/dbSNP, Pubmed, Google, etc) with supporting evidence (for example, that the effect of the variant is similar to WT) = **benign/likely benign.**

h. If the variant is recurrent in the MGL in-house database of subjects with different underlying diagnosis = **benign/likely benign.**

**NOTE:** For *TTN* synonymous variants will be classified as benign/likely benign.

For all options above, variant frequency information from the Exome Aggregation Consortium (ExAC browser, http://exac.broadinstitute.org/), was also obtained to assess available variant frequencies in various populations.

**SUPPLEMENTARY REFERENCES**

1. Celestino-Soper, P.B.S., et al., *Evaluation of the Genetic Basis of Familial Aggregation of Pacemaker Implantation by a Large Next Generation Sequencing Panel.* PloS one, 2015. **10**(12): p. e0143588.

2. Linderman, M.D., et al., *Analytical validation of whole exome and whole genome sequencing for clinical applications.* BMC Med Genomics, 2014. **7**: p. 20.

3. DePristo, M.A., et al., *A framework for variation discovery and genotyping using next-generation DNA sequencing data.* Nature genetics, 2011. **43**(5): p. 491-8.

4. Aziz, N., et al., *College of American Pathologists' laboratory standards for next-generation sequencing clinical tests.* Archives of pathology & laboratory medicine, 2015. **139**(4): p. 481-93.

5. Kent, W.J., et al., *The human genome browser at UCSC.* Genome Res, 2002. **12**(6): p. 996-1006.

6. Shihab, H.A., et al., *Predicting the functional, molecular, and phenotypic consequences of amino acid substitutions using hidden Markov models.* Human mutation, 2013. **34**(1): p. 57-65.

7. Klaassen, S., et al., *Mutations in sarcomere protein genes in left ventricular noncompaction.* Circulation, 2008. **117**(22): p. 2893-901.

8. Hershberger, R.E., et al., *Coding sequence mutations identified in MYH7, TNNT2, SCN5A, CSRP3, LBD3, and TCAP from 313 patients with familial or idiopathic dilated cardiomyopathy.* Clin Transl Sci, 2008. **1**(1): p. 21-6.

9. Richard, P., et al., *Hypertrophic cardiomyopathy: distribution of disease genes, spectrum of mutations, and implications for a molecular diagnosis strategy.* Circulation, 2003. **107**(17): p. 2227-32.

10. Brion, M., et al., *Sarcomeric gene mutations in sudden infant death syndrome (SIDS).* Forensic science international, 2012. **219**(1-3): p. 278-81.

11. Vatta, M., et al., *Mutations in Cypher/ZASP in patients with dilated cardiomyopathy and left ventricular non-compaction.* Journal of the American College of Cardiology, 2003. **42**(11): p. 2014-27.

12. Martinelli, V.C., et al., *ZASP interacts with the mechanosensing protein Ankrd2 and p53 in the signalling network of striated muscle.* PloS one, 2014. **9**(3): p. e92259.

13. Xi, Y., et al., *Loss of function of hNav1.5 by a ZASP1 mutation associated with intraventricular conduction disturbances in left ventricular noncompaction.* Circulation. Arrhythmia and electrophysiology, 2012. **5**(5): p. 1017-26.

14. Tan, B.-H., et al., *Common human SCN5A polymorphisms have altered electrophysiology when expressed in Q1077 splice variants.* Heart rhythm : the official journal of the Heart Rhythm Society, 2005. **2**(7): p. 741-7.

15. Gui, J., et al., *Mutation-specific effects of polymorphism H558R in SCN5A-related sick sinus syndrome.* J Cardiovasc Electrophysiol, 2010. **21**(5): p. 564-73.

16. Geisterfer-Lowrance, A.A., et al., *A molecular basis for familial hypertrophic cardiomyopathy: a beta cardiac myosin heavy chain gene missense mutation.* Cell, 1990. **62**(5): p. 999-1006.

17. Dausse, E., et al., *Familial hypertrophic cardiomyopathy. Microsatellite haplotyping and identification of a hot spot for mutations in the beta-myosin heavy chain gene.* The Journal of clinical investigation, 1993. **92**(6): p. 2807-13.

18. Spindler, M., et al., *Diastolic dysfunction and altered energetics in the alphaMHC403/+ mouse model of familial hypertrophic cardiomyopathy.* The Journal of clinical investigation, 1998. **101**(8): p. 1775-83.

19. Belus, A., et al., *The familial hypertrophic cardiomyopathy-associated myosin mutation R403Q accelerates tension generation and relaxation of human cardiac myofibrils.* The Journal of physiology, 2008. **586**(Pt 15): p. 3639-44.

20. Liu, W., C. Qian, and U. Francke, *Silent mutation induces exon skipping of fibrillin-1 gene in Marfan syndrome.* Nature genetics, 1997. **16**(4): p. 328-9.

21. Li, D., et al., *Novel cardiac troponin T mutation as a cause of familial dilated cardiomyopathy.* Circulation, 2001. **104**(18): p. 2188-93.

22. Lu, Q.-W., et al., *Cardiac troponin T mutation R141W found in dilated cardiomyopathy stabilizes the troponin T-tropomyosin interaction and causes a Ca2+ desensitization.* Journal of molecular and cellular cardiology, 2003. **35**(12): p. 1421-7.

23. Juan, F., et al., *The changes of the cardiac structure and function in cTnTR141W transgenic mice.* Int J Cardiol, 2008. **128**(1): p. 83-90.

24. Morita, H., et al., *Single-gene mutations and increased left ventricular wall thickness in the community: the Framingham Heart Study.* Circulation, 2006. **113**(23): p. 2697-705.

25. Merlo, M., et al., *Poor prognosis of rare sarcomeric gene variants in patients with dilated cardiomyopathy.* Clinical and translational science, 2013. **6**(6): p. 424-8.

26. Saltzman, A.J., et al., *Short communication: the cardiac myosin binding protein C Arg502Trp mutation: a common cause of hypertrophic cardiomyopathy.* Circulation research, 2010. **106**(9): p. 1549-52.

27. Van Driest, S.L., et al., *Myosin binding protein C mutations and compound heterozygosity in hypertrophic cardiomyopathy.* Journal of the American College of Cardiology, 2004. **44**(9): p. 1903-10.

28. Syrris, P., et al., *Arrhythmogenic right ventricular dysplasia/cardiomyopathy associated with mutations in the desmosomal gene desmocollin-2.* American journal of human genetics, 2006. **79**(5): p. 978-84.

29. De Bortoli, M., et al., *The p.A897KfsX4 frameshift variation in desmocollin-2 is not a causative mutation in arrhythmogenic right ventricular cardiomyopathy.* European journal of human genetics : EJHG, 2010. **18**(7): p. 776-82.

30. Hackman, P., et al., *Truncating mutations in C-terminal titin may cause more severe tibial muscular dystrophy (TMD).* Neuromuscul Disord, 2008. **18**(12): p. 922-8.

31. Evilä, A., et al., *Atypical phenotypes in titinopathies explained by second titin mutations.* Annals of neurology, 2014. **75**(2): p. 230-40.

32. Ceyhan-Birsoy, O., et al., *Recessive truncating titin gene, TTN, mutations presenting as centronuclear myopathy.* Neurology, 2013. **81**(14): p. 1205-14.

33. Kapplinger, J.D., et al., *Distinguishing arrhythmogenic right ventricular cardiomyopathy/dysplasia-associated mutations from background genetic noise.* J Am Coll Cardiol, 2011. **57**(23): p. 2317-27.

34. Pugh, T.J., et al., *The landscape of genetic variation in dilated cardiomyopathy as surveyed by clinical DNA sequencing.* Genetics in medicine : official journal of the American College of Medical Genetics, 2014. **16**(8): p. 601-8.

35. Herman, D.S., et al., *Truncations of titin causing dilated cardiomyopathy.* N Engl J Med, 2012. **366**(7): p. 619-28.

36. Hinson, J.T., et al., *HEART DISEASE. Titin mutations in iPS cells define sarcomere insufficiency as a cause of dilated cardiomyopathy.* Science, 2015. **349**(6251): p. 982-6.

37. Boehringer, T., et al., *SCN5A mutations and polymorphisms in patients with ventricular fibrillation during acute myocardial infarction.* Molecular medicine reports, 2014. **10**(4): p. 2039-44.

38. Kwon, H.W., et al., *Long QT syndrome and dilated cardiomyopathy with SCN5A p.R1193Q polymorphism: cardioverter-defibrillator implantation at 27 months.* Pacing Clin Electrophysiol, 2012. **35**(8): p. e243-6.

39. Vatta, M., et al., *Genetic and biophysical basis of sudden unexplained nocturnal death syndrome (SUNDS), a disease allelic to Brugada syndrome.* Human molecular genetics, 2002. **11**(3): p. 337-45.

40. Wang, Q., et al., *The common SCN5A mutation R1193Q causes LQTS-type electrophysiological alterations of the cardiac sodium channel.* Journal of medical genetics, 2004. **41**(5): p. e66.

41. Hu, Q., et al., *Inflammatory destruction of elastic fibers in acquired cutis laxa is associated with missense alleles in the elastin and fibulin-5 genes.* J Invest Dermatol, 2006. **126**(2): p. 283-90.

42. Loeys, B., et al., *Genotype and phenotype analysis of 171 patients referred for molecular study of the fibrillin-1 gene FBN1 because of suspected Marfan syndrome.* Archives of internal medicine, 2001. **161**(20): p. 2447-54.

43. De Backer, J., et al., *Utility of molecular analyses in the exploration of extreme intrafamilial variability in the Marfan syndrome.* Clinical genetics, 2007. **72**(3): p. 188-98.

44. Smith, C.G., et al., *Exome resequencing identifies potential tumor-suppressor genes that predispose to colorectal cancer.* Human mutation, 2013. **34**(7): p. 1026-34.

45. Campbell, I.M., et al., *TGFBR2 deletion in a 20-month-old female with developmental delay and microcephaly.* American journal of medical genetics. Part A, 2011. **155A**(6): p. 1442-7.

46. Kozich, V. and J.P. Kraus, *Screening for mutations by expressing patient cDNA segments in E. coli: homocystinuria due to cystathionine beta-synthase deficiency.* Hum Mutat, 1992. **1**(2): p. 113-23.

47. Linnebank, M., et al., *Haplotyping of wild type and I278T alleles of the human cystathionine beta-synthase gene based on a cluster of novel SNPs in IVS12.* Human mutation, 2001. **17**(4): p. 350-1.

48. Hnizda, A., et al., *Cystathionine beta-synthase mutants exhibit changes in protein unfolding: conformational analysis of misfolded variants in crude cell extracts.* J Inherit Metab Dis, 2012. **35**(3): p. 469-77.

49. Gupta, S., et al., *Correction of cystathionine beta-synthase deficiency in mice by treatment with proteasome inhibitors.* Hum Mutat, 2013. **34**(8): p. 1085-93.

50. Wang, D.W., et al., *Cardiac sodium channel dysfunction in sudden infant death syndrome.* Circulation, 2007. **115**(3): p. 368-76.

51. Albert, C.M., et al., *Cardiac sodium channel gene variants and sudden cardiac death in women.* Circulation, 2008. **117**(1): p. 16-23.

52. Kelleher, C.M., et al., *A functional mutation in the terminal exon of elastin in severe, early-onset chronic obstructive pulmonary disease.* Am J Respir Cell Mol Biol, 2005. **33**(4): p. 355-62.

53. Cooper, D.N., P.D. Stenson, and N.A. Chuzhanova, *The Human Gene Mutation Database (HGMD) and its exploitation in the study of mutational mechanisms.* Curr Protoc Bioinformatics, 2006. **Chapter 1**: p. Unit 1 13.

54. Husainova, R.I., et al., *[Examination of structural changes in the transforming growth factor beta receptor 1 (TGFbetaR1) gene in patients with chronic heart failure].* Genetika, 2014. **50**(5): p. 611-8.

55. Kapa, S., et al., *Genetic testing for long-QT syndrome: distinguishing pathogenic mutations from benign variants.* Circulation, 2009. **120**(18): p. 1752-60.

56. Kapplinger, J.D., et al., *An international compendium of mutations in the SCN5A-encoded cardiac sodium channel in patients referred for Brugada syndrome genetic testing.* Heart Rhythm, 2010. **7**(1): p. 33-46.

57. Refaat, M.M., et al., *Genetic variation in the alternative splicing regulator RBM20 is associated with dilated cardiomyopathy.* Heart rhythm : the official journal of the Heart Rhythm Society, 2012. **9**(3): p. 390-6.

58. Guo, W., et al., *RBM20, a gene for hereditary cardiomyopathy, regulates titin splicing.* Nat Med, 2012. **18**(5): p. 766-73.

59. Napolitano, C., et al., *Genetic testing in the long QT syndrome: development and validation of an efficient approach to genotyping in clinical practice.* JAMA, 2005. **294**(23): p. 2975-80.

60. Maekawa, K., et al., *Genetic polymorphisms and haplotypes of the human cardiac sodium channel alpha subunit gene (SCN5A) in Japanese and their association with arrhythmia.* Annals of human genetics, 2005. **69**(Pt 4): p. 413-28.

61. Refsgaard, L., et al., *High prevalence of genetic variants previously associated with LQT syndrome in new exome data.* European journal of human genetics : EJHG, 2012. **20**(8): p. 905-8.

62. Hong, K., et al., *Concomitant Brugada-like and short QT electrocardiogram linked to SCN5A mutation.* Eur J Hum Genet, 2012. **20**(11): p. 1189-92.

# Websites

1000genomes browser (URL: http://www.ncbi.nlm.nih.gov/variation/tools/1000genomes/)

1000G project release (genotypic information for NA12003, NA19449, NA19982, NA19704, NA11931, NA11829, and NA06986) (URL: ftp://ftp-trace.ncbi.nih.gov/1000genomes/ftp/release/20110521/)

**Berkeley Drosophila Genome Project (URL:** http://www.fruitfly.org/seq_tools/splice.html)

Bioinformatics pipeline scripts (http://compbio.iupui.edu/group/6/pages/clinicalsequencing)

CLinVar (URL: http://www.ncbi.nlm.nih.gov/clinvar/)

dbSNP (URL: http://www.ncbi.nlm.nih.gov/SNP/)

Exome Aggregation Consortium (ExAC), Cambridge, MA (URL: http://exac.broadinstitute.org) [December, 2015].

Exome Sequencing Project (URL: http://evs.gs.washington.edu/EVS/)

GATK UnifiedGenotyper (URL: https://www.broadinstitute.org/gatk/gatkdocs/org_broadinstitute_gatk_tools_walkers_genotyper_UnifiedGenotyper.php#--heterozygosity)

Google (URL: https://www.google.com/)

Human Splicing Finder (URL: http://www.umd.be/HSF/)

Illumina (URL: http://www.illumina.com/)

NA12878 genotype information (URL: ftp://ftp.platinumgenomes.org/trio)

NA19240 genotype information (URL: ftp://ftp-trace.ncbi.nih.gov/1000genomes/ftp/pilot_data/release/2010_07/trio/snps/)

NetGene2 Server (URL: http://www.cbs.dtu.dk/services/NetGene2/)

Picard-tools-1.105 (URL: [http://picard.sourceforge.net](http://picard.sourceforge.net./))

Pubmed (URL: http://www.ncbi.nlm.nih.gov/pubmed)

UCSC browser (URL: http://genome.ucsc.edu/)

**Figure legends**

**SUPPLEMENTARY FIGURE 1: Bioinformatics pipeline flowchart.**
