# Supplementary figures and images for: Validation and Utilization of a Clinical Next-Generation Sequencing Panel for Selected Cardiovascular Disorders
Source: Front Cardiovasc Med. 2017 Mar 15;4:11. doi: 10.3389/fcvm.2017.00011 (PMC5350117; doi:10.3389/fcvm.2017.00011)

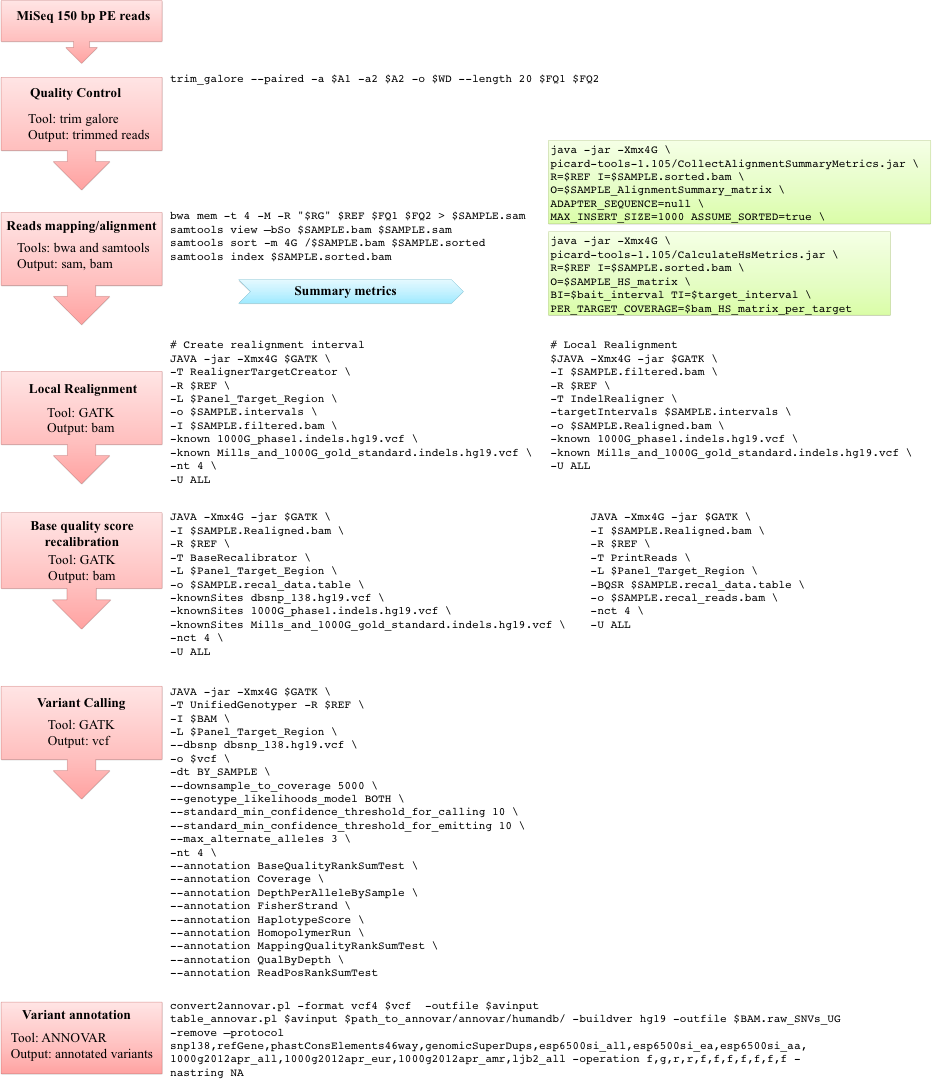

Supplement: Supplementary file 3 [file Image_1.TIF]
